# Comparison of air-liquid interface transwell and airway organoid models for human respiratory virus infection studies

**DOI:** 10.3389/fimmu.2025.1532144

**Published:** 2025-02-06

**Authors:** Camilla T. Ekanger, Nilima Dinesh Kumar, Rosanne W. Koutstaal, Fan Zhou, Martin Beukema, Joanna Waldock, Simon P. Jochems, Noa Mulder, Cécile A. C. M. van Els, Othmar G. Engelhardt, Nathalie Mantel, Kevin P. Buno, Karl Albert Brokstad, Agnete S. T. Engelsen, Rebecca J. Cox, Barbro N. Melgert, Anke L. W. Huckriede, Puck B. van Kasteren

**Affiliations:** ^1^ Department of Biomedicine, Faculty of Medicine, University of Bergen, Bergen, Norway; ^2^ Centre for Cancer Biomarkers (CCBIO), Department of Clinical Medicine, University of Bergen, Bergen, Norway; ^3^ Influenza Centre, Department of Clinical Science, University of Bergen, Bergen, Norway; ^4^ Department of Medical Microbiology and Infection Prevention, University Medical Center Groningen, University of Groningen, Groningen, Netherlands; ^5^ Center for Infectious Disease Control, National Institute for Public Health and the Environment (RIVM), Bilthoven, Netherlands; ^6^ Influenza Resource Centre, Vaccines, Science Research & Innovation, Medicines and Healthcare Products Regulatory Agency (MHRA), Potters Bar, United Kingdom; ^7^ Leiden University Center for Infectious Diseases (LU-CID), Leiden University Medical Center, Leiden, Netherlands; ^8^ Groningen Research Institute for Pharmacy, Department of Molecular Pharmacology, University of Groningen, Groningen, Netherlands; ^9^ Section Immunology, Department Biomolecular Health Sciences, Faculty of Veterinary Medicine, Utrecht University, Utrecht, Netherlands; ^10^ Sanofi, Marcy L’Etoile, France; ^11^ GlaxoSmithKline (GSK), Siena, Italy; ^12^ Department of Safety, Chemistry and Biomedical Laboratory Sciences, Western Norway University of Applied Sciences, Bergen, Norway; ^13^ Department of Microbiology, Haukeland University Hospital, Bergen, Norway; ^14^ Groningen Research Institute for Asthma and COPD, University Medical Center Groningen, University of Groningen, Groningen, Netherlands

**Keywords:** influenza virus, mucosal models, respiratory tract, complex *in vitro* models, harmonization

## Abstract

**Introduction:**

Complex *in vitro * respiratory models, including air-liquid interface (ALI) transwell cultures and airway organoids, have emerged as promising tools for studying human respiratory virus infections. These models address several limitations of conventional two-dimensional cell line and animal models. However, the lack of standardized protocols for the application of these models in infection studies limits the possibilities for comparing results across different research groups. Therefore, we applied a collaborative approach to harmonize several aspects of experimental methodology between different research laboratories, aiming to assess the comparability of different models of human airway epithelium in the context of respiratory viral infections.

**Methods:**

In this study, we compared three different models of human respiratory epithelium: a primary human bronchial epithelial cell-derived ALI transwell model, and two airway organoid models established from human airway- and lung-derived adult stem cells. We first assessed the presence of various differentiated cell types using immunofluorescence microscopy. Using a shared stock of influenza A virus, we then assessed viral growth kinetics, epithelial cytokine responses, and serum-mediated inhibition of infection.

**Results:**

The presence of club, goblet, and ciliated cells was confirmed in all models. We observed similar viral replication kinetics with a >4-log increase in virus titre across all models using a TCID50 assay. Following infection, a reproducible antiviral cytokine response, including a consistent increase in CXCL10, IL-6, IFN-λ1, IFN-λ2/3, and IFN-β, was detected across all models. Finally, neutralization was assessed by pre-incubation of virus with human serum. Reduced viral replication was observed across all models, resulting in a 3- to 6-log decrease in virus titres as quantified by TCID50.

**Discussion:**

In conclusion, all three models produced consistent results regardless of the varying cell sources, culturing approaches, and infection methods. Our collaborative efforts to harmonize infection experiments and compare ALI transwell and airway organoid models described here aid in advancing our understanding and improving the standardization of these complex *in vitro* respiratory models for future studies.

## Introduction

Respiratory viruses, including influenza virus, severe acute respiratory syndrome coronavirus 2 (SARS-CoV-2), and respiratory syncytial virus (RSV), are a considerable public health concern, imposing a significant global economic burden (1–3). While vaccines have been developed for several respiratory viruses, continuing efforts are aimed at improving vaccine efficacy and breadth (4–6). These efforts necessitate the use of representative preclinical models that accurately reflect the complexity of the respiratory epithelium. The respiratory system is divided into the upper respiratory tract - comprising the nose, nasal cavity, and pharynx - and the lower respiratory tract, which includes the trachea, bronchial tubes, and lungs (7). The conducting airway epithelium, lining the passages from the nasal cavity to the terminal bronchioles, is primarily composed of ciliated, goblet, club, and basal cells. Mucociliary clearance, driven by beating ciliated cells and mucus-producing goblet cells and mucus glands, plays an important role in immediate protection against inhaled pathogens, alongside the tissue-resident immune cells (8). Finally, the alveoli, which are the functional unit of the lungs, are populated mainly by type I and type II pneumocytes and provide the conditions required for gas exchange between the alveolar air sacs and the capillaries.

In preclinical vaccine development, animal models, traditional 2D cell culture models, and complex *in vitro* models are utilized. Animal models (e.g., murine, ferret, and non-human primate models) serve as a crucial tool for mimicking the pathophysiology of viral disease and are valuable for testing vaccine-induced immunogenicity and protection (9–12). However, despite their usefulness, animal models are costly to develop and maintain, raise ethical concerns (hence 3Rs), and the clinical relevance of animal models in human virology research is limited by inherent differences between species. Conventional cell line-based models commonly used for virus isolation and propagation offer several advantages, including readily available reagents, suitability for high-throughput assays, and the existence of well-established protocols and pipelines. However, traditional 2D cell cultures are often based on animal-derived cell lines with genetic abnormalities and are generally homogenous, lacking the cellular diversity and differentiation characterizing the human respiratory epithelium. In addition, these cultures lack a clear apical-basal polarity, which is critical for accurate modelling of viral entry and replication.

Transitioning from conventional approaches, the Inno4Vac consortium (www.inno4vac.eu) aims to accelerate vaccine research by further developing *in silico*, *in vivo*, and *in vitro* infection models to study vaccine responses and protection. One pillar of this collaborative and interdisciplinary project focuses on bridging the gap between traditional 2D cell cultures and *in vivo* animal models by exploring the use of complex *in vitro* mucosal models for different organ systems, including the digestive, urogenital, and respiratory systems. Developing vaccines using these complex mucosal models may shorten vaccine testing procedures. This subsequently reduces the gap between preclinical and clinical models and the reliance on 2D cell cultures and *in vivo* animal models.

**Figure 1:**
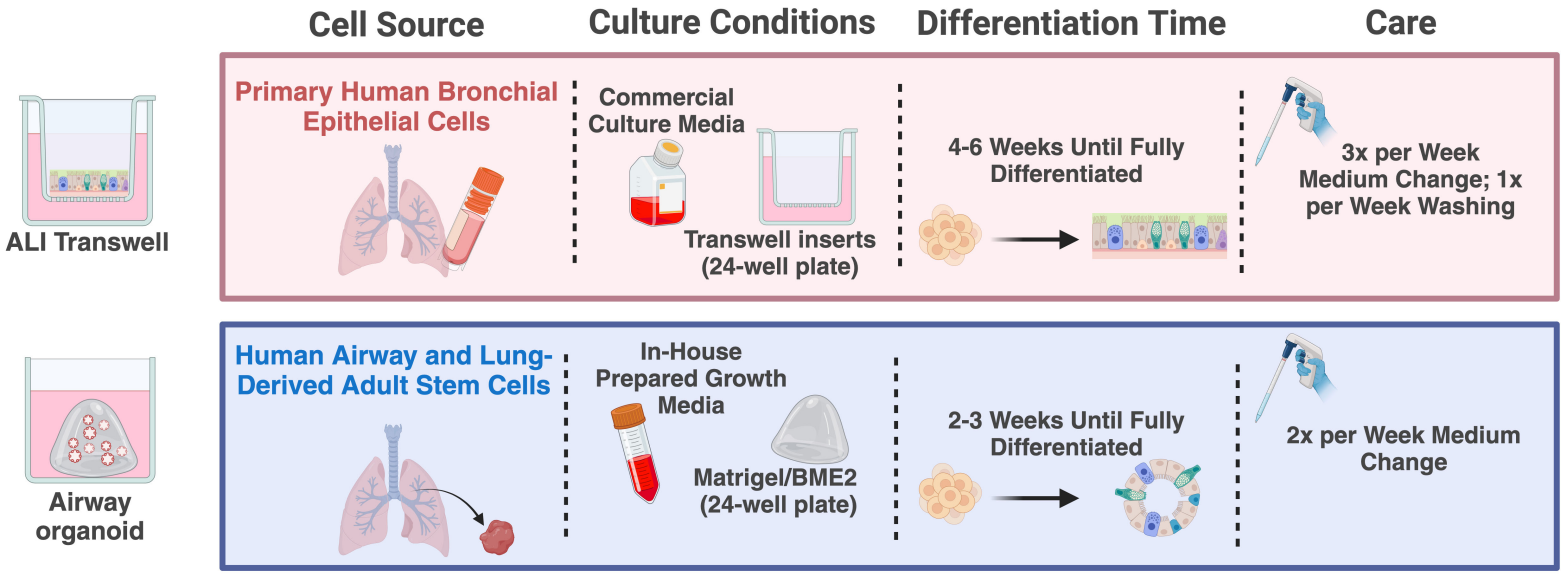
Schematic overview of culturing procedures for the ALI transwell and airway organoid models. Created in BioRender (https://BioRender.com/e98t944).

The widely used air-liquid interface (ALI) transwell model is established from primary human airway epithelial cells derived from the nasal, proximal, or distal airway epithelium (13–15). These cells are cultured on transwell inserts with a porous membrane, with essential nutrients and differentiation factors supplied to the cells through the medium on the basal side (15, 16). The distinctive feature of the ALI transwell model lies in exposing the apical side of the cells to the air, creating an “air-liquid interface” or ALI (**Figure 1**). In 4-6 weeks, the ALI transwell cultures develop into fully differentiated, pseudostratified airway epithelium. This differentiated epithelium displays several characteristics of human respiratory epithelium, such as beating ciliated cells, mucus-producing goblet cells, and tight junctions forming a protective barrier. The ALI transwell model´s close resemblance to the human airway environment, its capability for prolonged cultivation, and its spatially defined infection interface make it a suitable system for the study of host-pathogen interaction in a controlled artificial environment, yet closely mimicking physiological conditions (17).

Human airway organoids have emerged as a complementary system for studying respiratory virus infections (18–21). Organoids are complex *in vitro* 3D cultures established from sources such as organ-derived adult stem cells or induced pluripotent stem cells. Cultivating these cells in an extracellular matrix, such as Matrigel or basement membrane extract (BME2) gel, combined with a growth factor-enriched medium, induces a process that stimulates a proliferation and differentiation process resulting in organoids that mimic the functional characteristics of the human respiratory epithelium (20, 21). Following 2-3 weeks in culture, the features of the airway organoids include several hallmarks of the human airway epithelium, such as the dynamic beating of cilia and mucus production from goblet cells, along with the presence of basal and club cells. A key feature of this model is the orientation of the apical side of the epithelium, facing toward a hollow lumen (**Figure 1**). This orientation inherently restricts immediate virus access to the apical side of the epithelium, which usually contains the virus-relevant receptors. To expose the apical side of the epithelium to infection, enzymatic dissociation or mechanical disruption of the organoid structures are commonly used strategies (18, 22).

Over the last decade, the use of ALI transwell and airway organoid models has increased. However, the absence of standardized protocols within this field limits the possibilities for assessing the comparability of results obtained with different models. Therefore, in the current Inno4Vac study, we harmonized several aspects of our experimental approach, including using a shared virus stock, influenza A/Wisconsin/588/2019 (H1N1)pdm09, and applying the same sampling time points and experimental readouts, to compare characteristics of three different models across three research groups: a primary human bronchial epithelial cell-derived ALI transwell model and two airway organoid models established from human airway- and lung-derived adult stem cells, respectively. We confirmed the presence of relevant cell types, assessed viral replication kinetics and antiviral cytokine responses, and explored antibody-mediated virus neutralization in all models. Overall, our results reveal comparability between the different models, suggesting that all models are suitable for virus infection research, with the choice depending largely on the specific research question to be addressed.

## Materials and methods

### Primary cell acquisition and epithelial model culturing procedures

Each model was cultured according to the procedures previously established within each of the research groups, which are detailed below. A schematic overview of the culturing procedures can be found in **Figure 1**.

#### Air-liquid interface transwell model

Primary human bronchial epithelial cells (HBEC) from healthy donors (Lonza, #CC-2540, Switzerland) were expanded in complete PneumaCult Ex Plus medium (Stemcell Technologies, #05040, Canada) until the cells reached log phase. Cells were then harvested using trypsin (Lonza, #CC-5012), which was inactivated using trypsin neutralizing solution (Lonza, #CC-5002). Passage 1 cells were frozen in 78% Ham’s nutrient mix F12 (Merck/Sigma, #51651C), 10% heat-inactivated foetal bovine serum (FBS), 2% 1.5 M HEPES (pH 7.2), and 10% DMSO, and stored at -135°C until further use.

Stored cells were thawed in Complete PneumaCult Ex Plus medium in a T75 culture flask and expanded until log phase was reached, replacing the medium every 1-2 days. The cells were harvested using trypsin as described above and seeded at a density of 9x10^4^ cells/well on the apical side of transwell inserts with a PET membrane with pore size 0.4 μm and diameter 6.5 mm (Corning, #CLS3470, USA) in 100 μL PneumaCult Ex Plus medium. In addition, 650 μL culture medium was added to the basolateral compartment. After 48-72 h, confluent monolayers were airlifted by aspirating the media from both compartments and adding 500 μL complete PneumaCult ALI medium (Stemcell Technologies, #05001) supplemented with 100 units/mL Penicillin and 0.1 mg/mL Streptomycin (Pen/Strep, Sigma, P4333-100mL), 0.48 μg/mL Hydrocortisone (Stemcell Technologies, #07926) and 4 μg/mL Heparin solution (Stemcell Technologies, #07980), to the basolateral compartment only. Medium was replaced every 2-3 days (500 μL/well for 2 days, 650 μL/well for 3 days of culture). From week 2 post airlift, the apical side of the cells was washed 1-2 times per week using 200 μL PBS (Gibco, #20012-068, USA) to remove excess mucus. Cells were kept in a humidified incubator at 37°C and 5% CO_2_.

#### Matrigel-cultured airway organoid model

In this study, the patient-derived airway organoids were established from lung tissue-derived human adult stem cells obtained from lung resections of non-small cell lung cancer patients (n = 3) based on the method developed by Sachs et al., with modifications previously described by Ekanger and colleagues (20, 21). Briefly, minced fragments of human lung tissue were prepared using a pair of surgical scalpels (Swann-Morton, #0507, UK). Subsequently, the minced tissue was carefully transferred into a 50 mL Falcon tube using a cell lifter (VWR, #76036-004, USA). 10 mL of AdDF+++ washing medium (AdDF (Gibco, 12634028) supplemented with 10 mM HEPES (Gibco, 15630106), 1% Pen/Strep, and 1x GlutaMax (Gibco, 35050061)) was used to wash the minced tissue, followed by centrifugation at 400 xg for 5 min. The minced lung tissue was resuspended in AdDF+++ washing medium, containing 2 mg/mL collagenase (Sigma-Aldrich, #C9407), and subjected to an orbital shaker, maintaining a temperature of 37°C for 2 h. After the enzymatic digestion, the remaining tissue fragments were sheared using a 10 mL Pasteur pipette and filtered through a pre-wetted 100 μm filter (Corning, #431752, USA). The resulting strained suspension was collected, and 2% FBS was added before a 400 xg centrifugation for 5 min. To eliminate residual erythrocytes, 2 mL of red blood cell lysis buffer (Roche, #11814389001, Germany) was added to the pellet for 5 min at RT. Finally, 10 mL AdDF+++ washing medium was added, and the suspension was centrifuged at 400 xg for 5 min.

Lung cell pellets were resuspended in ice-cold Growth Factor Reduced Matrigel (Corning, #356231), where approximately 12,500 human lung epithelial cells per 50 μL droplets of Matrigel were seeded onto 37°C pre-warmed 24-well plates (SARSTEDT, #83.3922, Germany). To prevent cells from sinking to the bottom and adhering to the plastic surface, the plates were incubated in an inverted orientation at 37°C for 30 min to allow solidification of the Matrigel. Upon solidification, droplets were immersed in 400-500 μL per well of organoid medium A (**Table 1**). To enhance the efficiency of primary human organoid formation, the organoid medium was supplemented with 5 µM of ROCK inhibitor Y-27632 (Stemcell Technologies, #72304), for a period of 4 days from the start of culturing. The cultures were placed in a humidified 5% CO_2_, 5% O_2_ incubator set at 37°C. To sustain optimal growth conditions, the organoid medium was changed every 4 days. Passaging of the organoids was carried out every 2-3 weeks, depending on the growth rate and confluence of the culture, as previously described by Ekanger and colleagues (21). For cryopreservation, the serum-free cell freezing medium Bambanker Direct (Genetics, #BB01, USA) was used, as described (21).

**Table 1:** Organoid medium components.

	Concentration	Medium A (Matrigel organoids)	Medium B (BME2 organoids)
**AdDF medium**	N/A	Gibco, 12634028	Gibco, 12634028
**R-Spondin 1**	500 ng/mL	Peprotech, #120-38	Peprotech, #120-38
**Human FGF-7**	25 ng/mL	Peprotech, #100-19	Peprotech, #100-19
**Human FGF-10**	100 ng/mL	Peprotech, #100-26	Peprotech, #100-26
**Noggin**	100 ng/mL	Peprotech, #120-10C	Peprotech, #120-10C
**A83-01**	500 nM	Tocris, #2939	Tocris, #2939
**SB202190**	500 nM	Sigma, #S7067	Sigma, #S7067
**B27 supplement**	1x	Gibco, #17504044	Gibco, #17504044
**N-Acetylcysteine**	1.25 mM	Sigma, #A9165	Sigma, #A9165
**Nicotinamide**	5 mM	Sigma, #N0636	Sigma, #N0636
**GlutaMax**	1x	Invitrogen, #12634-034	Gibco, #3505006
**HEPES**	10 mM	Invitrogen, #15630-056	Gibco, #15630056
**Pen/Strep**	100 U/mL	Invitrogen, #15140-122	Gibco, #15070063
**Amphotericin B**	2.5 µg/mL	Sigma, #A294	–
**Primocin**	100 µg/mL	–	Invitrogen, #ant-pm-05
**Y-27632** ^a^	5 µM	Stemcell Technologies, #72304	Axon Medchem, #1683

a ROCK inhibitor Y-27632 was only included for a period of 4 days from the start of culturing in the Matrigel-organoid medium.

#### BME2-cultured airway organoid model

Tissues were obtained from lung transplant donors post-mortem, from residual tracheal and main stem bronchial tissues, after a lung donation, and stored in Krebs buffer (117.5 mM NaCl; 5.6 mM KCl; 1.18 mM MgSO4 · 7 H2O; 1.28 mM NaH2PO4 · H2O; 2.52 mM CaCl2 · 2 H2O; 25 mM NaHCO3; 5.55 mM D-glucose· H2O in sterile H2O) at 10°C prior to epithelial cell isolation. Selection criteria for transplant donors are listed in the Eurotransplant guidelines and include the absence of primary lung disease, such as asthma and COPD, and no more than 20 pack years of smoking history.

After excision, the tissues were trimmed, washed, and incubated overnight at 4°C in PBS supplemented with 200 U/mL of Pen, 200 μg/mL of Strep (Gibco, #15070-063) and 10 μg/mL of the anti-mycoplasma agent ciprofloxacin (Sigma, #17850). Tracheal rings were then incubated for 2 h at 37°C in Ca2^+^- and Mg2^+^-free Hanks’ balanced salt solution (HBSS; Lonza, #14175) supplemented with Pen/Strep and 0.18 mg/mL of protease XIV (Sigma, #P5147, 4P8). Epithelial cells were gently scraped off the luminal surface, washed twice, and subsequently cultured in keratinocyte serum-free medium (KSFM; Gibco, #17005-034) on six-well plates (Greiner, #657970) that were precoated for 2–6 h with a combination of 30 μg/mL PureCol (Advanced BioMatrixSan Diego, #5005-B), 10 μg/mL fibronectin (Sigma, #F0895), and 10 μg/mL bovine serum albumin (BSA; Sigma, #A2153) in PBS. KSFM was supplemented with 0.2 ng/mL of epidermal growth factor (EGF; Gibco, #17005-075), 25 μg/mL of bovine pituitary extract (BPE; Gibco, #17005-075), 1 μM isoproterenol (Sigma, #I-6504), 200 U/mL Pen, and 200 μg/mL Strep. During the first week of culture, the culture medium was supplemented with the anti-mycoplasma agent ciprofloxacin. After 90% confluency was reached, the cells were trypsinized using 0.5 g/L trypsin, 0.2 g/L EDTA (Lonza, #BE17-161E), and either cultured for a maximum of two subsequent passages or cryopreserved and stored in liquid nitrogen. For cryopreservation, cells were suspended in KSFM medium supplemented with BPE at a concentration of 0.3 mg/mL and 10% DMSO.

Per well, 20,000 freshly isolated epithelial cells were resuspended in 50 μL Cultrex growth factor-reduced Basement Membrane Extract (BME2, R&D, #3533- 010-02). Droplets of 50 μL BME2-cell mixture were added on pre-warmed 24-well suspension culture plates (Greiner, #M9312) at 37°C for 30 min. After solidification, droplets were immersed in 500 μL per well of organoid medium B (**Table 1**). Cultures were incubated at 37°C in a humid incubator containing ambient air, supplemented with 5% CO_2_ for 3 weeks. The organoid medium in the wells was refreshed every 3-4 days.

### Immunofluorescence staining

#### Air-liquid interface transwell model

Both sides of the transwell membrane were washed twice with PBS, after which the cells were fixed in 4% formaldehyde (VWR, #9713.5000) on both sides, for at least 30 min at 4°C. After fixation, the membranes were washed three times with PBS (Gibco, #20012-068). Cells were blocked and permeabilized using 0.5% Triton X-100 (CAS #9036-19-5, Merck Millipore #108603) in PBS (PBST 0.5%) with 5% donkey serum (Abcam, #ab7475). Cells were stained with rat anti-α-Tubulin (1:500; Invitrogen, #MA1-80017), rabbit anti-Muc5AC (1:100; Abcam, #Ab198294), goat anti ZO-1 (1:250; Invitrogen, #PA5-19090), mouse anti-CC10 (1:250; SantaCruz Biotech, #Sc-365992) and/or mouse anti-influenza NP (1:1000; Abcam, #Ab20343) in PBS with 5% donkey serum for 1 h at RT. Membranes were washed three times with PBST 0.1% before adding the secondary antibody mix for 1 h at RT. All secondary antibodies were donkey-anti mouse/rat/rabbit/goat conjugated to AF405 plus, AF488 plus, AF555 plus, or AF647 plus (Invitrogen), used at a dilution of 1:800.

Membranes were washed three times with PBST 0.1% and one final time with PBS. The membranes were excised using a scalpel and mounted on glass slides using ProLong Diamond Antifade Mountant (Invitrogen, #P36961). Slides were left to dry o/n and the edges were sealed with clear nail polish. Imaging was done using a Nikon A1R confocal laser scanning microscope at the Centre for Cell Imaging (Utrecht University, NL) and further analysis was done using FIJI software. The confocal microscopy maximum projection image shown is composed of 15 images obtained with a Z-step of 0,22 μm obtained in a maximum 49,9 μm thick stack of the specimen.

#### Matrigel-cultured organoid model

For immunofluorescent staining, organoids were extracted from Matrigel by adding 1 mL ice-cold AdDF+++ washing medium to each well. The organoids were transferred to a 15 mL Falcon tube and centrifuged at 400 xg for 5 min, then washed once with PBS and fixed at RT for 24 h using 3.7% formaldehyde (Sigma Aldrich, #252549, USA). After fixation, organoids were centrifuged at 400 xg for 5 min, then stained with 1% aqueous methyl green (Sigma-Aldrich, #M8884) for 2 min. Subsequently, the organoids were resuspended in approximately 50-100 μL 60°C pre-heated 1.5% low gelling temperature agarose (Sigma- Aldrich, #A9045) in Tris-Buffered Saline (TBS) and transferred to a 60°C pre-heated 50 mL Falcon tube. To ensure even distribution at the bottom of the Falcon tube, the organoids suspended in agarose were centrifuged for 1 min at 400 xg before agarose solidification. The tube was then placed at 4°C for 20-30 min to allow the agarose to solidify. Next, the agarose-embedded organoids were transferred to a cell-safe biopsy capsule (Cell Path, #EBE-0201-02A, UK) and immersed in 70% ethanol for preservation until paraffin embedding and sectioning, according to standard procedures. Briefly, formalin-fixed and paraffin-embedded (FFPE) organoids were cut using a microtome to produce sections of 6 μm thickness, which were collected on SuperFrost+ object-glasses followed by deparaffinization, using a sequential immersion method: xylene (5 min + 10 min), 99% ethanol (2x 5 min), 96% ethanol (2x 5 min), 70% ethanol (5 min), 50% ethanol (5 min), ddH20 (2x 5 min). Antigen retrieval was then performed through a 30 min heat treatment at 95°C in DAKO Target Retrieval Solution (Agilent, #S1699, USA) using Decloaking Chamber NxGen (Biocare Medical, USA).

Organoid sections were subsequently blocked in TBS-Tween-20 containing 1% BSA (Sigma-Aldrich, #A1470) for 30 min, before undergoing o/n incubation at 4°C with primary antibodies, including pan-cytokeratin (1:200; Agilent, #M3515), ARL13B (1:400; Proteintech, #17711-1AP), MUC5AC (1:400; Cell Signalling Technology, #6119), CC10 protein (1:200; Santa Cruz, #sc-365992), Keratin 5 (1:200; BioLegend, #905901), and anti-Influenza A H1N1 Virus Nucleoprotein antibody (1:100; Bio-Techne, #NBP2-16965). The following day, organoid sections were washed three times in TBS-Tween, before being incubated with secondary antibodies for 1 h at RT. These included goat anti-rabbit, Alexa fluor 647 (Invitrogen, #A21244), goat anti-mouse, Alexa fluor 546 (Invitrogen, #A11029), and goat anti-chicken, Alexa fluor 488 (Jackson, #122-605-167). Nuclear staining was achieved by staining with 0.25 μg/mL 4′,6-diamidino-2-phenylindole (DAPI) for 2 min. Finally, organoid sections were mounted with 5-10 μL Prolong Diamond mounting medium (Thermo Fisher, #P36961, USA), and 18 x 18 mm confocal coverslips were placed on the specimens (Zeiss, Oberkochen, Germany). Images were taken using the Axio Vert microscope (Zeiss, Germany) or Eclipse Ti2 (Nikon, Japan). Scale bars were added using FIJI software.

#### BME2-cultured organoid model

To harvest organoids from the BME2 gel, 1 mL of cold AdDF+++ washing medium was added to each well. After centrifugation at 500 xg for 5 min, the organoids were washed once with cold AdDF+++ medium. The resulting organoid pellet was then suspended in 500 μL of 3.7% paraformaldehyde (PFA; Brunschwig, #J61899) for fixation at RT for 15 min, followed by two PBS washes. Subsequently, the organoids were suspended in 120 µL of warm HistoGel (65°C, Thermo Fisher, #R904012) and allowed to solidify as droplets for 30 min before placing into cassettes which are then transferred to 70% ethanol. The HistoGel droplet with organoids was embedded in paraffin (Epredia, USA) following standard protocol and cut into 3 µm sections using the MicroM HM 340E machine (Thermo Fisher, USA) which were collected on glass microscope slides (Klinipath, USA). Deparaffinization was performed using xylene (2x 5 min), 99% ethanol (2x 20 dips), 96% ethanol (2x 20 dips), 70% ethanol (1x 20 dips), ddH20 (1x 20 dips) and washed three times in PBS. After deparaffinization, antigen retrieval was carried out by heating the sections in citric acid (10 mM, pH 6.0) followed by three PBS washes. Sections were then incubated in PBST 0.5% for 10 min, followed by three additional PBS washes. Next, sections were blocked in 5% BSA and 5% normal human serum (NHS; Invitrogen, #31876) in PBS for 15 min. Subsequently, the sections were incubated overnight at 4°C with the following primary antibodies diluted in 2% BSA with 1% NHS in PBS: ARL13B (1:400; Proteintech, #17711-1AP), MUC5AC (1:400; Cell Signalling Technology, #6119), CC10 (1:250; Santa Cruz, #sc365992), p63 (1:2000; Abcam, #ab124672), followed by three PBS washes. The samples were then blocked with a buffer of 5% BSA with 5% normal goat serum (NGS; Invitrogen, #31873) in PBS followed by incubation for 2 h at RT with the following secondary antibodies diluted in 2% BSA and 1% NHS in PBS: goat anti-rabbit Alexa fluor 647 (Invitrogen, #A21244), goat anti-mouse Alexa Fluor 568 (Invitrogen, #A11031) and goat anti-rabbit Alexa Fluor 568 (Invitrogen, #A1101). Specimens were mounted in Prolong Gold antifade reagent with DAPI (Invitrogen, #P36931) before adding coverslips. Images were acquired using the Ti2 microscope (Nikon, Japan). Scale bars were added using FIJI software.

For influenza nucleoprotein staining, PFA-fixed organoids were not embedded in paraffin, but were instead collected in a 1.5 mL Eppendorf tube. The staining procedure was performed as described above, with a centrifugation step for 5 min at 300 xg in between each step. Samples were stained with the primary Influenza A H1N1 Nucleoprotein Antibody (1:2000; Novus Biologicals, #NBP2-16965, USA). During incubation with the secondary antibody, samples were stained with goat anti-rabbit Alexa Fluor 568 (1:300, Invitrogen, #A1101) and Alexa Fluor™ 488 Phalloidin (1:400, Invitrogen, #A12379). Organoids were suspended in 50 μl of Prolong Gold antifade reagent with DAPI, before adding to a glass microscope slide (Klinipath).

### Shared virus stock and titration

#### Stock production

Influenza A/Wisconsin/588/2019 (H1N1)pdm09 (from the Medicines and Healthcare products Regulatory Agency [MHRA] repository, UK) was propagated on Madin-Darby Canine Kidney (MDCK, ATCC CCL-34) cells seeded in T175 flasks, to reach 80-90% confluency on the day of the infection. Virus was diluted 1:500 in serum-free Ultra-MDCK medium (Biowhittaker, #BE12-749Q) supplemented with 1% pen/strep (Gibco, #15140-122) and 2 µg/mL TPCK-trypsin (Sigma, #T1426), and 3 mL of virus-containing medium was added to each flask. Following a 1 h incubation at 35°C, the inoculum was removed and 50 mL of medium was added to each flask. The flasks were incubated at 35°C with 5% CO_2_ for 4 days, until a cytopathic effect of >80% was observed. The content of the flasks was collected and centrifuged for 10 min at 900 xg to spin down cellular debris. The supernatant was harvested, aliquoted, stored at -70°C, titrated and distributed among the different research groups involved in the project.

#### Virus stock titration

Virus titres were determined by 50% tissue culture infective dose (TCID50) assay. Briefly, 100µl of 1 X 10^5^ ml^-1^ MDCK cells were added to each well of a 96 well plate and incubated overnight at 37°C with 5% CO_2_. Virus serial dilutions (10^-3^ to 10^-10^) were prepared in DMEM, supplemented with L-glut, pen/strep/amphotericin, HEPES, non-essential amino acids (NEAAs), 0.2% BSA and 2 μg/ml of TPCK-trypsin, transferred to washed MDCKs in 96‐well culture plates (including 10 replicates per dilution) and incubated at 35°C with 5% CO_2_ for 45 min. Inoculum was then replaced with 100 µl medium per well and incubated for 3 days at 35°C with 5% CO_2_. Virus presence was detected using a hemagglutination (HA) test by transferring 50 µL of the supernatant to V‐bottom 96‐well plates and adding 50 µL of turkey red blood cell suspension (at a concentration of 0.7% in PBS) to each well. Following a 30 min incubation at RT, the number of hemagglutinated wells was scored for each virus dilution, and the TCID50 titre was determined using the Spearman & Kärber method.

### Viral infection and sampling procedures

#### Air-liquid interface transwell model

To ensure full differentiation and the presence of mature cilia before starting infection experiments, HBEC cultures were visually monitored for mucus production and ciliary movement. Based upon these observations, infection experiments were performed at 5-7 weeks post airlifting. Three days before infection, the basal cell culture medium was replaced with complete PneumaCult ALI medium without heparin and hydrocortisone (ALI infection medium). Directly before infection, cells were washed apically using 200 μL/well HBSS (Gibco, #14025092) for 10 min at 37°C, and the basal medium was replaced with 500 µL ALI infection medium/well. Influenza virus was diluted to 5x10^4^ TCID50/mL in HBSS. Cells were infected apically in triplicate using 200 μL inoculum, corresponding to 1x10^4^ TCID50 per well, and incubated at 37°C and 5% CO_2_ for 1 h, after which the inoculum was aspirated. Cells were immediately washed thrice with 200 μL HBSS. For virus quantification, apical washes were performed at 2, 24, 48, and 72 hours post infection (hpi) by adding 200 μL HBSS and incubating 15 min at 37°C. Samples were snap-frozen using 100% EtOH in dry ice and stored at -80°C until further analysis. In parallel with the apical washes, 50 μL basolateral medium samples were obtained from each well for cytokine analysis. These samples were also snap-frozen and stored at -80°C.

Virus quantification was performed by TCID50 assay on MDCK cells. In brief, samples were serially diluted 1:12 in OptiMEM (Gibco, #11520386), supplemented with 100 units/mL Pen and 0.1 mg/mL Strep (Sigma, #P4333). Virus dilutions were added in quadruplicate to a confluent monolayer of MDCK cells, and incubated at 37°C for 5 days. Subsequently, each well was scored either negative or positive for cytopathic effects, and viral load was determined using the Spearman & Kärber method.

#### Matrigel-cultured organoid model

Organoids were cultured in organoid medium A for 2-3 weeks before virus infection. The medium was removed and 2 mL/well ice cold AdDF+++ washing medium was added to dissolve the Matrigel. The organoids were pooled and transferred to a 15-mL falcon tube, resuspended in 2 mL TrypLE (Gibco, #12604013) for dissociation, and incubated no longer than 10 min at 37°C. Then, 5 mL of AdDF+++ washing medium was added to the dissociated organoids. Cells were counted at this step to allow for MOI calculations, using the Countess 3 Automated Cell Counter (Invitrogen, #AMQAX2000). The enzymatically dissociated organoids were centrifuged at 400 xg for 5 min and the pellet was incubated with the virus inoculum, diluted in AdDF+++ medium, at a MOI of 0.1, for 1 h at 37°C in an incubator on a shaker. The organoids were washed twice with AdDF+++ washing medium and transferred to a 24-well ultra-low attachment plate (Corning, #3473) in a volume of 500 μL organoid medium A per well. When collecting the virus-containing supernatant at 2, 24, 48, or 72 hpi, organoids were mechanically sheared to release the virus from the organoid lumen into the culture medium. No additional medium was added to the wells and the suspension was centrifuged at 400 xg for 5 min. 50 μL of the virus-containing supernatant was collected from each sample to determine cytokine levels. The remaining supernatant was used to determine the virus titre. Finally, the organoid pellet was fixed by adding 3.7% formaldehyde for 24h at RT, followed by FFPE processing.

Virus titres were determined by TCID50 assay on MDCK-SIAT1 cells (RRID: CVCL_Z936). In brief, an undiluted sample followed by an 8-step, 10-fold serial dilution was used, using HA at 72 h as the readout. Titration was performed with 6 replicates, and the titre was calculated using the Reed-Muench method.

#### BME2-cultured organoid model

Following 3 weeks of culture, the organoid medium was removed, and 1 mL of ice cold AdDF+++ washing medium was added to each well. The differentiated organoids were mechanically sheared by passing them 25-30 times through a 10 µL tip attached to a 1 mL pipette to expose the apical/luminal surface. The sheared organoids were washed once with AdDF+++ and centrifuged at 500 xg for 5 min. The resulting pellet was incubated with the virus inoculum at a MOI of 0.01 in 250 µL of AdDF+++ medium containing 3 µg/mL TPCK-trypsin for 1 h at 37°C. The organoids were then washed twice with AdDF+++ medium and resuspended in 500 µL organoid medium B, supplemented with 3 µg/mL of TPCK-trypsin, and placed in suspension in 24-well plates pre-treated with anti-adherence Rinsing Solution (Stemcell Technologies, #07010). At 2, 24, and 48 hpi, organoids were mechanically sheared to release the virus from the organoid lumen into the culture medium. The suspension was centrifuged at 500 xg for 5 min to collect the virus-containing supernatant. The supernatant samples were snap-frozen in liquid nitrogen and stored at -80°C.

Virus titres were determined using a TCID50-based HA assay. Confluent MDCK cells, grown in Episerf serum-free medium (Gibco, #10732-022), were inoculated with 100 µL of 10-fold serial dilutions of the virus suspension with titrations performed in duplicate or quadruplicate. After 1 hour, the plates were washed once with PBS, and 150 µL of Episerf medium containing TPCK trypsin (Sigma, #T1426) was added to reach a final concentration of 5 µg/mL. After 72 h, the supernatants were collected and transferred to a V-bottom 96-well plate (Corning, #3898), and 50 μL of 1.5% guinea pig erythrocytes (Inotiv, West Lafayette, USA) were added to each well. The plate was incubated for 2 h, and wells were scored for positive HA. The Spearman & Kärber method was used to calculate the TCID50.

To determine the cell numbers for MOI calculation, the organoid medium was removed and 1 mL of ice-cold AdDF+++ medium was added to break and collect the matrix-containing organoids. Organoids were pelleted by centrifuging at 500 xg for 5 min and organoids from 1 or 2 wells were dissociated into single cells by resuspension in 2 mL TrypLE (Invitrogen) followed by incubation for 10-15 min at 37°C in a water bath. Cells were resuspended in AdDF+++ and centrifuged at 500 xg for 5 min. The pellet was resuspended in 1 or 2 mL of AdDF+++ and counted to determine the total number of cells per well.

### Serum neutralization assay

The same human serum pool was used for the virus neutralization assay on all models. Hemagglutination-inhibition titre and neutralizing titre of a serum panel were determined, and the sera with the highest titres were pooled and distributed among research groups. Pooled serum was assessed in a dilution of 1:80 and 1:400 on each model.

#### Air-liquid interface transwell model

The preparation of the well-differentiated HBEC cultures was performed according to the procedure described above for the viral infection kinetics experiments. Diluted serum was mixed at a 1:1 ratio with influenza virus at 1x10^6^ TCID50/mL in HBSS and incubated for 2 h at 37°C. For the control condition, the virus was mixed 1:1 with HBSS. Before infection, the cells were washed apically with 200 µL HBSS for 10 min at 37°C. 200 µL of the virus/serum solution was added to the apical side of the transwells in triplicate, followed by a 1 h incubation at 37°C. Subsequently, the ALI cultures were apically washed 3 times with 200 µL HBSS to remove the inoculum. The cells were incubated at 37°C, and apical washes were collected at 24 hpi as previously described, to monitor viral replication.

#### Matrigel-cultured airway organoid model

Influenza virus at an MOI of 0.1 was incubated with diluted serum for 1 h at 37°C. Infection and sampling of enzymatically disrupted organoids was carried out as described above.

#### BME2-cultured airway organoid model

Serum dilutions in AdDF+++ were mixed at a 1:1 ratio with influenza virus at an MOI of 0.01 and incubated for 1 h at 37°C. For the control condition, the virus was mixed 1:1 with AdDF+++ medium. Infection and sampling of mechanically disrupted organoids was carried out as described above.

### Multiplex cytokine analysis

Cytokine concentrations were determined using the LEGENDplex™ Human Anti-Virus Response Panel (Biolegend, #740390) according to the manufacturer’s instructions, except for the use of a reduced volume per reaction. This panel contained the following cytokines: IL-1β, IL-6, CXCL8 (IL-8), IL-10, IL- 12p70, IFN-α2, IFN-β, IFN-λ1 (IL-29), IFN-λ2/3 (IL-28a/b), IFN-γ, TNF-α, CXCL10 (IP-10), GM-CSF. Only those cytokines that were consistently detected are shown.

## Results

### ALI transwell and airway organoid models display subtle differences in histoarchitecture, cellular differentiation and composition

**Figure 2:**
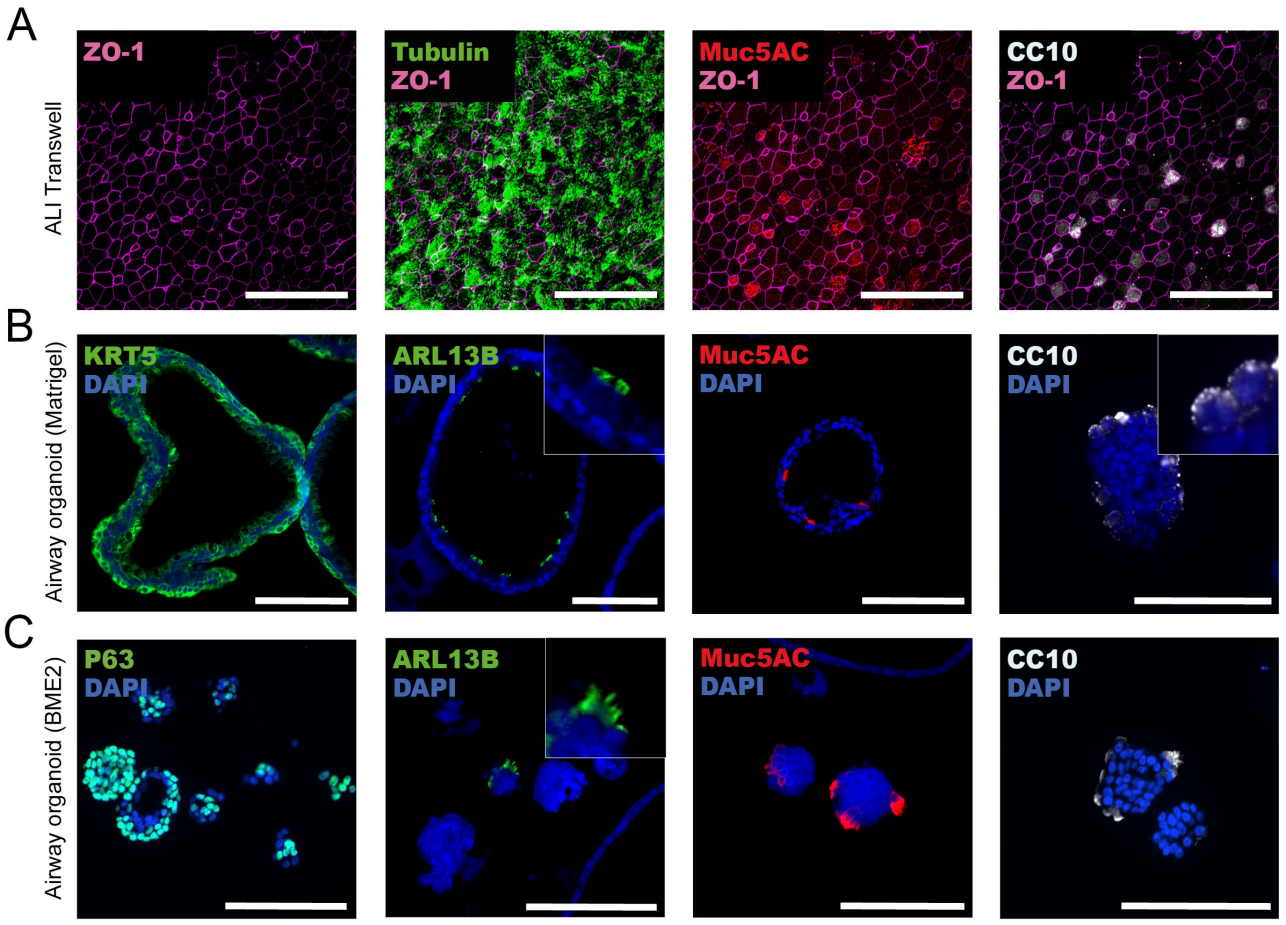
Characterization of histoarchitecture and cell type composition of air-liquid interface (ALI) transwell and airway organoid models. **(A)** Representative images from one donor of ALI transwell cultures displaying immunofluorescent staining of tight junctions (ZO-1), ciliated cells (α tubulin), secretory goblet cells (MUC5AC), and club cells (CC10). The maximum projection image shown is composed of 15 confocal images acquired with a Z-step of 0.22 μm, giving a total thickness of 3.3 μm. Scale bars equal 50 μm. **(B, C)** Representative immunofluorescent images from well-differentiated airway organoids cultured in Matrigel **(B)** or BME2 **(C)**. Immunofluorescent labelling of stained formalin-fixed and paraffin-embedded sections of organoids showing the expression of markers for basal cells (KRT5/P63), cilia (ARL13B), secretory goblet cells (MUC5AC), and club cells (CC10). Scale bars equal 100 μm.

We characterized the histoarchitecture of the ALI transwell and airway organoid models, and the presence of the cell types characteristic of the human respiratory airway epithelium (**Figure 2**). After 4-6 weeks in culture, the ALI transwell model displayed the distinctive features of pseudostratified airway epithelium, including mucus production and ciliary beating. By microscopy of immunofluorescently stained formaldehyde-fixed transwell inserts, secretory goblet cells were identified by mucin 5AC (MUC5AC) expression, club cells were identified by Clara cell 10kDa protein (CC10) expression, and ciliated cells were identified by α tubulin expression (**Figure 2A**). Basal cells could not readily be identified by immunofluorescent staining of epithelium from the ALI transwell model. Immunofluorescent staining of tight junction protein Zonula Occludens-1 (ZO-1) was included to provide a clear delineation of the cell boundaries (**Figure 2A**).

In the adult stem cell-derived airway organoid models cultured in Matrigel or BME2, differentiated airway epithelium was formed after 2 or 3 weeks of culture, respectively. The airway organoids cultured in Matrigel gradually enlarged and formed a prominent hollow lumen (**Figure 2B**). We have previously shown that after 13 days in culture, the airway organoids had an average diameter of approximately 200 μm (21). In contrast, the BME2 cultures had fewer organoids forming a hollow lumen, and most organoids remained smaller, with an average diameter ranging from 50 to 100 µM (**Figure 2C**). In the airway organoid models, immunofluorescent staining was performed on FFPE tissue sections to assess the presence of the four major cell types of the conducting airways of the human respiratory system: basal, ciliated, goblet, and club cells. Basal cells were identified in both the airway organoids cultured in Matrigel and BME2 using two interchangeable basal cell markers, keratin 5 (KRT5) and P63, respectively. In BME2 organoids, P63-positive cells were localized centrally in smaller and less differentiated organoids. However, in organoids with a well-defined hollow lumen, P63-positive cells were positioned with a characteristic basal location at the periphery of the organoids (**Figure 2C**). ADP-ribosylation factor-like protein 13B (ARL13B) plays a role in cilia formation and maintenance and, as expected, expression of ARL13B was located in mature cilia [**Figures 2B, C** (zoomed inserts)]. ARL13B-expressing cells facing the lumen of the organoids were detected in Matrigel organoids, whereas in BME2-cultured organoids, these cells were mostly observed on the outer layer (**Figures 2B, C**). MUC5AC-expressing cells were detected in both airway organoid models, with an apical orientation towards the lumen of the organoids cultured in Matrigel (**Figure 2B**). Production and secretion of mucosubstances into the lumen have previously been shown for this organoid model by alcian-blue periodic acid Schiff’s histochemistry (21). In the BME2 organoids, the localization of MUC5AC-positive cells depends on whether a hollow lumen is formed. In organoids with a hollow lumen, MUC5AC was observed with an apical orientation towards the lumen. Conversely, when a hollow lumen was not formed, MUC5AC appeared to be located on cells in the outermost layers of the spheres (**Figure 2C**). In airway organoids grown in Matrigel and BME2, CC10-containing vacuoles in club cells were frequently detected in the outermost layers of the organoids (**Figures 2B, C**).

### ALI transwell and airway organoid models are similarly susceptible to influenza A/H1N1 virus infection and viral replication

Next, we examined the infectivity and replication of influenza A/Wisconsin/588/2019 (H1N1)pdm09 virus in human ALI transwell and airway organoid models, derived from a total of 9 individual human donors. All infection experiments were performed using aliquots of the same virus stock, and each model was established from cells of 3 independent donors. Differentiated primary human bronchial epithelial cells in the ALI model were infected with the influenza A/H1N1 virus at 5-7 weeks post-airlifting. For virus quantification, apical washes were performed with 3 technical replicates (inserts) per time point for each donor. The airway organoids cultured in Matrigel or BME2 were infected with influenza A/H1N1 after 2-3 weeks of differentiation. To facilitate viral access to sialic acids on the apical side of the epithelial cells, organoids were enzymatically (Matrigel) or mechanically (BME2) disrupted before infection. At the indicated time points, the airway organoids were mechanically sheared to release the virus from the lumen of re-assembled organoids into the culture medium prior to harvest.

**Figure 3:**
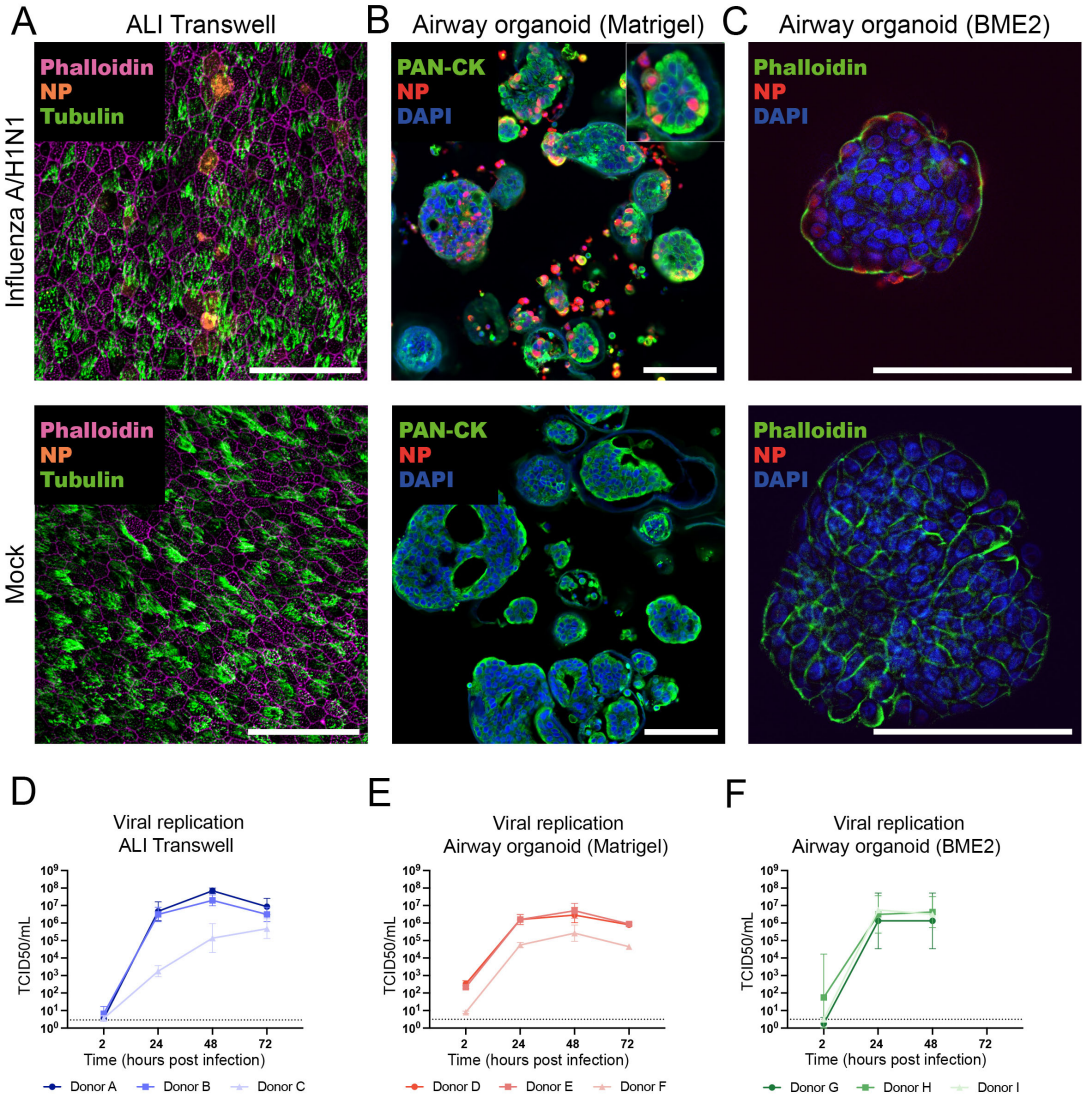
Comparison of influenza A/H1N1 virus infection susceptibility and infection kinetics of airway air-liquid interface (ALI) transwell and organoid models. **(A)** At 24 hpi, infected ALI transwell cultures were immunostained to detect viral nucleoprotein (NP, orange), ciliated cells (α-tubulin, green), and actin filaments (phalloidin, purple). Scale bar equals 50 μm. **(B, C)** At 24 hpi, infected organoids cultured in Matrigel **(B)** or BME2 **(C)** were visualized by immunostaining of formalin-fixed and paraffin-embedded sections (for Matrigel) and non-paraffin-embedded organoids (for BME2) to detect viral nucleoprotein (NP, red), actin filaments were stained with pan-cytokeratin (PAN-CK, green) or phalloidin (green), and nuclei were stained by DAPI (blue). Scale bars equal 100 μm. **(D–F)** Virus quantification was performed by TCID50 assay on Madin-Darby Canine Kidney (MDCK) cells for all 3 models. TCID50/ml for the time points 2, 24, 48, and 72 hpi are shown. **(D)** At the indicated time points, apical washes were collected from infected ALI transwell cultures (MOI=0.01) from 3 donors (A/B/C). The data shown are from 3 technical replicates (inserts) per donor. **(E)** Human airway organoids derived from 3 donors (D/E/F) were cultured in Matrigel, infected following enzymatic dissociation (MOI=0.1), and sampled at the indicated hpi. The data shown are from 2 technical replicates (wells) per donor. **(F)** Organoids derived from 3 donors (G/H/I) were cultured in BME2, infected with influenza virus following mechanical disruption (MOI=0.01), and sampled at the indicated hpi. The data shown are from 1 (I) or 2 (G/H) technical replicates (wells) per donor. Replicates were obtained in 2 independent experiments. The limit of detection was 2.89 TCID50/mL for panel **(D)** and 3.16 TCID50/mL for panels **(E, F)**. The geometric mean with geometric standard deviation (SD) is shown.

To confirm the presence of influenza virus protein in epithelial cells, infected ALI transwell cultures and organoids were fixed at 24 hpi and immunostained to identify influenza A virus nucleoprotein (NP) in infected cells. Representative immunofluorescent micrographs from one donor per model confirmed influenza A/H1N1 infection and NP expression in both ALI transwell and airway organoid cultures grown in Matrigel and BME2 (**Figures 3A–C**). To compare infection kinetics between the models, a virus titration assay (50% tissue-culture infectious dose; TCID50) was performed on MDCK cells using samples harvested from the infected ALI transwell and airway organoids at different time points up to 72 hpi. The virus titre increased by at least 3-log for all donors across all models by 48 hpi and plateaued at 72 hpi (**Figures 3D–F**).

### ALI transwell and airway organoid cultures display similar cytokine release profiles upon influenza A/H1N1 virus infection

**Figure 4:**
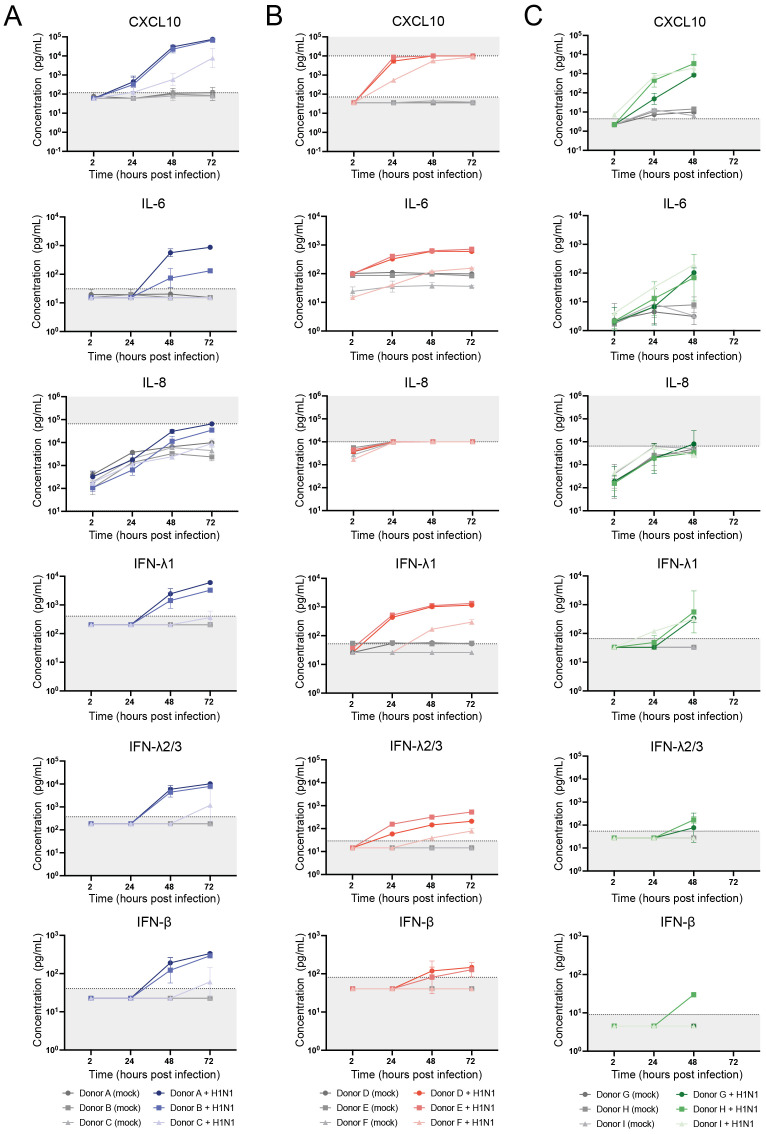
Dynamics of host responses to A/H1N1 influenza virus infection in air-liquid interface (ALI) transwell and airway organoid models, measured by cytokine production. Concentrations of the cytokines CXCL10, IL-6, IL-8, IFN-λ1, IFN-λ2/3, and IFN-β were measured using LEGENDplex assay. **(A)** Basolateral medium samples from (mock-)infected ALI transwell cultures of 3 donors (A/B/C) were collected at the indicated time points and assessed for cytokine production. The data shown are from 3 technical replicates (inserts) per donor. **(B)** Cytokine concentrations were measured in the supernatant of (mock-)infected dissociated airway organoids cultured in Matrigel, derived from 3 donors (D/E/F). The data shown are from 2 technical replicates (wells) per donor. **(C)** Cytokine concentrations were measured in the supernatant of (mock-)infected dissociated airway organoids cultured in BME2 from 3 donors (G/H/I). The data shown are from 1 (I) or 2 (G/H) technical replicates (wells) per donor. Replicates were obtained in 2 independent experiments. The geometric mean with geometric SD is shown. For visualization purposes, cytokine concentrations below the limit of quantification (LOQ) were assigned values of 0.5 times the LOQ. Dotted lines and grey shading indicate the LOQ for each cytokine.

In response to a respiratory virus infection, airway epithelium is known to secrete inflammatory cytokines to alert immune cells of the invading pathogen. To compare the host response dynamics to influenza A/H1N1 virus between the different models, we measured the production of 13 cytokines (IL-1β, IL-6, IL-8, IL-10, IL- 12p70, IFN-α2, IFN-β, IFN-λ1, IFN-λ2/3, IFN-γ, TNF-α, CXCL10, GM-CSF) using a multiplex immunoassay. For ALI transwell cultures, cytokine-containing medium samples were collected from the basolateral side. In contrast, for the airway organoid models, the medium collection was performed after mechanical disruption to release cytokines secreted from the apical side of the epithelium into the lumen of the organoids. Thus, samples harvested from the airway organoid models may include both basally- and apically-secreted cytokines. Among the 13 cytokines measured, CXCL10, IL-6, IL-8, IFN-λ1, IFN-λ2/3, and IFN-β were consistently detected in A/H1N1 virus-infected samples across all models (**Figures 4A–C**). Cytokine concentrations increased in a time-dependent manner, with overall higher concentrations in the infected samples compared to the mock-infected controls, except for IL-8 for which the measured concentrations were similar between the two conditions in the organoid models, possibly because the upper limit of quantification of the assay was reached.

### Human serum inhibits viral infection in ALI transwell and airway organoid cultures

**Figure 5:**
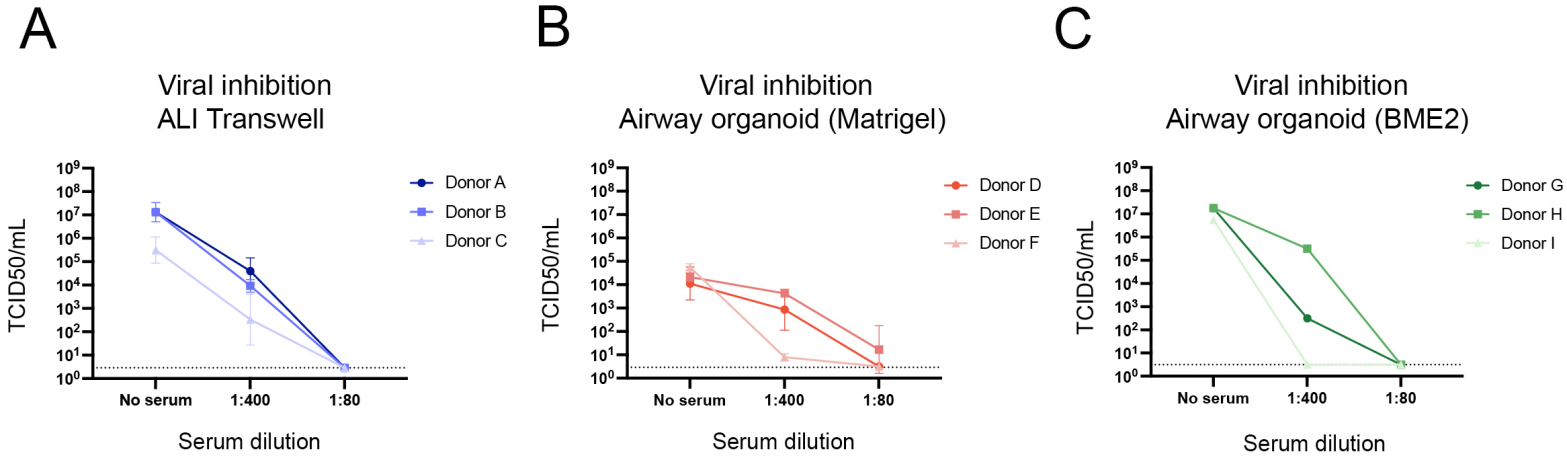
Inhibition of viral replication by pooled human serum containing influenza virus-specific neutralizing antibodies. TCID50/mL values at 24 hours post infection are shown for all models for three conditions: no serum, 1:400 dilution of serum, and 1:80 dilution of serum. **(A)** Values are from ALI transwell cultures from 3 donors (A/B/C). The data shown are from 3 technical replicates (inserts) per donor. **(B)** Values from airway organoids cultured in Matrigel derived from 3 donors (D/E/F) are shown. The data shown are from 2 technical replicates (wells) per donor. **(C)** Values from airway organoids cultured in BME2 derived from 3 donors (G/H/I), without technical replicates. For all 3 models, virus quantification was performed by TCID50 assay on Madin-Darby Canine Kidney cells, where the limit of detection was 2.89 TCID50/mL for panel **(A)** and 3.16 TCID50/mL for panels **(B, C)**. The geometric mean with geometric standard deviation (SD) is shown **(A, B)**.

To explore the potential for assessing antibody-mediated neutralization of virus infection in the models, a pool of human serum collected from individuals vaccinated with the 2022/23 seasonal influenza vaccine was tested at dilutions of 1:80 and 1:400. Influenza A/H1N1 virus was pre-incubated with this serum pool before being added to ALI transwell or dissociated airway organoid cultures. At 24 hpi, viral titres exhibited a marked reduction compared to the no serum controls across all models (**Figures 5A–C**). Specifically, a serum dilution of 1:80 achieved complete neutralization of infection in both ALI transwell and airway organoid cultures. In contrast, a serum dilution of 1:400 resulted in partial neutralization in most cultures, except for organoids derived from donors F and I, for which complete neutralization was also achieved at this dilution (**Figures 5B, C**). These data indicate that human serum can effectively inhibit viral replication in both ALI transwell and airway organoid models, and that the models are relevant for evaluating antibody-mediated neutralization of virus infection.

## Discussion

The aim of the current study was to compare different models of human respiratory epithelium in the context of viral infection. To facilitate this evaluation, several essential experimental aspects were harmonized across three independent laboratories of the Inno4Vac consortium. Harmonization steps included using the same virus stock, influenza A/Wisconsin/588/2019 (H1N1)pdm09, and pooled human serum for assessing antibody-mediated neutralization of virus infection in the models. Harmonization further included aligning sampling time points and employing similar methods for quantifying uniform readouts for comparison. To capture the inter-laboratory heterogeneity/comparability of these complex *in vitro* models, the source of primary cells and differentiation protocols were excluded from harmonization efforts. Our results suggest that the ALI transwell and airway organoid models, in general, provide comparable outcomes regarding virus infection kinetics and host response to infection, with subtle differences in the cell type composition and tissue differentiation status across the models that serve to highlight how the models may complement each other.

Each of the models was cultured according to standard procedures previously established and implemented by each of the three research groups. Based on previous experience, the ALI transwell inserts were deemed ready for infection following 5-7 weeks of differentiation, whereas the airway organoid models were allowed a shorter generation and differentiation time of 2-3 weeks before proceeding to infection. Notably, at the time of infection, basal cells were observed in both airway organoid models, while immunofluorescent staining could not readily identify basal cells in the ALI transwell cultures. Although not assessed in the current study, previous studies have reported a gradual loss of basal cells in ALI transwell models, potentially due to the differentiation of these cells into other cell types, most prominently ciliated cells (23, 24).

The results from our studies suggest that the two airway organoid models evaluated represent tissues of varying degrees of differentiation. BME2 organoids appeared to be less mature compared to Matrigel-based organoids, as suggested by the smaller size, fewer differentiated ciliated cells and reduced presence of a hollow lumen and a clear apical-basal orientation of tissues in the former. One explanation for the observed differences between BME2 and Matrigel organoids could be the choice of extracellular matrix material and culture medium composition, which are both known to influence organoid maturation and lumen formation (25). Most notably, ROCK inhibitor was continuously present in the culture medium of the BME2 organoids, whereas it was removed after 4 days of culturing for the Matrigel organoids. ROCK inhibitor is generally applied transiently after thawing or passaging to allow cell recovery and increase airway organoid formation efficiency. Additionally, the source of tissue used for cell isolation varied between the two airway organoid models. In the case of Matrigel organoids, cells were isolated from whole lung tissue, whereas for BME2 organoids, cells were derived specifically from the tracheal ring. These differences in the cellular starting material could further contribute to the observed disparities in organoid development, potentially reflecting distinct cellular responses to their respective microenvironments or variations in cell populations present in the two tissue sources. The ability to modify the experimental protocol to produce airway organoids of various differentiation statuses may be considered an advantage since it allows researchers to address various research questions related to tissue maturation and pathophysiology affecting tissue histoarchitecture and differentiation.

The source of cells used for modelling human respiratory epithelium requires careful consideration. In the current study, the primary cells used for the generation of differentiated epithelial models were obtained from different sources, either commercially obtained for the ALI transwell model or in-house collected and from slightly different anatomical locations for the airway organoid models. Most likely, each of these cell sources can be used interchangeably for the generation of each of the described models. Assessing the impact of applying the same starting material in the various differentiation protocols would be an interesting question for future studies. Notably, each primary cell source possesses its own advantages and limitations, which may influence the selection of a particular route of acquisition. For example, commercially available primary cells are readily available from various providers and require little time and effort for preparation. In contrast, in-house derived primary cells require ethical permissions and interdisciplinary collaborations between several departments in clinical institutions, as well as considerable effort and expertise for successful isolation and propagation. However, in-house isolation may come with the advantage of being able to obtain more background information on the donors and higher numbers of cells that allow for longer-term use of models generated from cells isolated from the same donor.

The current study focused on human *in vitro* models that recapitulate the histoarchitecture and cellular composition of tissues of the conducting airways. Importantly, epithelial models for additional anatomical regions of the respiratory tract have been developed, allowing for the study of respiratory virus infection in areas such as the upper airway epithelium. Protocols now exist that use non-invasive techniques to develop human nasal organoids and ALI transwell cultures from nasal epithelial cells, rather than relying on biopsy samples (26, 27). This approach is beneficial as the sampling procedure takes place in a readily accessible site, allowing for a relatively simple in-house collection of primary cells, albeit in low numbers. In addition, both organoid and lung-on-chip models of the alveolar epithelium of the lung have been developed (28–30). Together, these different models of various anatomical sites of the respiratory organ system provide researchers with the appropriate toolbox that is essential to investigate specific mechanisms of infection, histopathology, and protection at various anatomical locations, bridging one of the gaps between conventional cell lines and animal and human infection models.

While performing this study, we noticed the difficulties researchers encounter when attempting to harmonize an experimental set-up between different model systems used by different research groups, especially when techniques have already been established within laboratories. While we succeeded in aligning various aspects of our experiments and analyses, including the harmonization of sampling time points, readouts, and – arguably most importantly – the virus stock, divergence in other areas remained. As mentioned above, each research group used primary cells from the source they were accustomed to in the generation of models. Consequently, the experiments performed for each model included a different set of donors. However, despite the use of different primary cell sources and donors, our results provide a strong indication that, in general, these models are quite comparable. Considering a research landscape in which it will be challenging to harmonize every single aspect, these results provide some reassurance to the research community that valuable insights can nonetheless be obtained across models. In addition, it highlights the need for characterization of models and a detailed description of the experimental procedures in each study to allow reproducibility.

Another limitation of our study is the fact that we only assessed a single virus in our experiments. The obtained results thus serve as proof-of-concept to demonstrate that each of the models in our study may support respiratory virus replication to a similar extent, resulting in a similar pattern of cytokine secretion. However, highly pathogenic influenza strains or virus species that more strongly depend on the presence of specific receptors for infection, such as RSV and SARS-CoV-2 (31–33), might reveal more subtle differences in susceptibility and host response between the different models. Such potential differences might also depend on the cell type composition and maturation status of the model, which in the current study was only assessed at a single endpoint and using a limited number of biological replicates (3 donors). Additional research is needed to address these points in more depth.

A distinct feature of commonly used airway organoids is their apical-in polarity. In this arrangement, the apical side of the epithelium, where virus attachment receptors are generally located (18), faces inward toward the lumen. In contrast, the basal side is oriented outward, facing the supportive extracellular matrix. This apical-in polarity limits the immediate access of virus to the apical surface of the epithelium. For this reason, methods such as microinjection into the lumen of the organoids or enzymatic and mechanical organoid disruption are necessary to ensure virus access to the apical side of the organoids and to allow sampling of apically-secreted cytokines. Differences in the method of organoid disruption affects cellular composition and morphology, and may affect infection efficiency, as has previously been reviewed by Aguilar et al. (34). Recently, experimental protocols have been developed to generate apical-out airway organoids, which may represent a more efficient and scalable method for respiratory virus infection (19), also because these protocols do not include the use of an extracellular matrix. In contrast to airway organoids, the readily exposed apical side of the epithelium of the ALI transwell model allows for more straightforward virus inoculation and targeted sampling of the apical and basolateral sides separately, without the need for additional manipulation.

An attractive next step for the use of complex *in vitro* models in preclinical vaccine development will be the incorporation of both humoral and cellular immune components. This would result in an even more representative model of the protective mucosal barrier. In this study, we have confirmed that pre-incubation of virus with serum, as expected, prevented infection in all three models. In the future, a more accurate mimic of the response to viral infection *in situ* could be achieved by pre-conditioning the epithelial models with virus-specific antibodies obtained from sources such as serum or mucosal lining fluid. In addition, the co-culture of epithelial models with immune cells could provide further insights into additional immunological processes involved in protection from infection. The cytokines not shown in this study (IL-1β, IL-10, IL- 12p70, IFN-α2, IFN-γ, TNF-α, GM-CSF) are known to be mainly produced by cells not currently present in our models. The addition of immune cell to the epithelial airway models would be expected to induce a broader and more potent cytokine response, with a cytokine profile more similar to the response detected following viral infection in a clinical setting. Of note, the increased complexity of the models upon the addition of several non-epithelial cellular components would benefit from analyses by high-dimensional imaging modalities to evaluate the cell-type specific kinetics of cytokine production.

Whereas the apical-in nature of traditional organoids and the possibility of culturing in suspension allows for the relatively straightforward addition of immune cells to the basal side of the epithelium (i.e. the side where blood-derived immune cells enter the epithelium *in vivo*), the physical properties of the ALI transwell model pose a somewhat greater barrier. Firstly, the physical distance between the epithelium resting on the transwell insert and immune cells located at the bottom of the culture well of the basolateral compartment precludes direct physical interaction between the two. A possible solution entails a modification of the protocol where the epithelial cells are cultured on the underside of the transwell membrane insert, with growth media and immune cells within the transwell insert. However, the actual migration of immune cells towards the epithelial layer remains hampered by the small pore size of the transwell insert membrane. Epithelial cell differentiation on membranes with larger pore sizes remains technically challenging and might require the inclusion of fibroblasts and/or endothelial cells, adding yet another layer of complexity to the model. Considering that endothelial cells have already been shown to influence the epithelial cytokine response upon influenza virus infection (35), this might however be an essential step towards the optimal mucosal infection model.

In conclusion, collaborative efforts, such as the one described here, are crucial for a better understanding of the use of complex *in vitro* models of human respiratory epithelium and to support efforts in standardizing protocols and ensuring the reproducibility of results. Each of the complementary models described here offers distinct advantages and limitations, including possibilities for sampling and the amount of expertise and hands-on time required for culturing. The optimal research model should be selected carefully based on the specific scientific question to be addressed. Despite their inherent differences, the models evaluated in this study displayed considerable comparability in viral replication kinetics, host responses to infection, and the effect of human serum on infection, supporting their added value for pre-clinical vaccine research.

## Data Availability

The raw data supporting the conclusions of this article will be made available by the authors, without undue reservation.
